# Comparison of Initial Psychological Treatment Selections by US and Japanese Early-Career Psychiatrists for Patients with Major Depression: A Case Vignette Study

**DOI:** 10.1007/s40596-015-0398-6

**Published:** 2015-08-13

**Authors:** Aya Williams, Atsuo Nakagawa, Mitsuhiro Sado, Daisuke Fujisawa, David Mischoulon, Felicia Smith, Masaru Mimura, Yuji Sato

**Affiliations:** University of California, Berkeley, Berkeley, CA USA; Keio University School of Medicine, Tokyo, Japan; Massachusetts General Hospital, Boston, MA USA

**Keywords:** Major depressive disorder, Psychotherapy, Treatment selection, Residency, Culture

## Abstract

**Objective:**

The authors compared early-career psychiatrists’ selection of psychological treatments for patients with mild to moderate major depressive disorder (MDD) in the US and Japan.

**Methods:**

A total of 120 early-career psychiatrists from two residency programs in the US and Japan participated in web-based surveys. The psychiatrists selected first- and second-line psychological treatments in response to two case vignettes of patients with mild and moderate MDD.

**Results:**

Eighty-one psychiatrists (68 %) returned the surveys, of whom 39 (48 %) were American and 42 (52 %) Japanese. In response to the mild MDD case, more US psychiatrists selected high-intensity psychological treatments (*P* < 0.001), whereas more Japanese psychiatrists selected low-intensity psychological treatments (*P* < 0.001). In both countries, more psychiatrists selected psychological treatment than medication. In response to the moderate MDD case, one third of the US psychiatrists selected high-intensity psychological treatments (*P* < 0.001), whereas half of the Japanese psychiatrists selected low-intensity psychological treatments (*P* = 0.010).

**Conclusions:**

Residency training, availability of psychological treatments, and cultural beliefs may shape differences in treatment selections, which in turn may impact the dissemination and implementation of psychological treatment in clinical practice across cultures.

Although various psychological treatments have been shown to be effective for the treatment of major depressive disorder (MDD), few are readily available in clinical practice today, even in high-income countries [1]. Although approximately twice as many patients with MDD prefer psychological treatments to medication [2], large-scale household surveys have indicated that only a small fraction of patients with common mental health disorders in the community are offered evidence-based psychotherapy treatments [3]. Data from approximately 600 clinicians in independent practice indicate that they primarily rely on past clinical experience rather than empirical research to inform treatment decisions, illustrating a wide and enduring research-practice gap in the dissemination and implementation of psychological treatments [4].

According to McHugh and Barlow [1], the greatest challenge in disseminating and implementing evidence-based psychological treatments is training clinicians to competently administer them. This training can be particularly difficult due to the complex and nuanced nature of psychotherapy. Successful training in psychological treatments involves both didactic and competency training; the former requires information transfer through written materials, lectures, and workshops, and the latter requires acquisition of the skills necessary to administer treatment with fidelity [1]. Because didactic training alone is insufficient to create sustainable changes in clinical practice, recent attention has been focused on competency training in the form of clinical supervision [5].

In psychiatry, residency programs in the US and Japan have similarly sought to increase psychological treatment competency in early-career psychiatrists, including residents. Psychiatrists in the US are required to achieve competency in low-intensity psychological treatment, such as non-directive supportive therapy, as well as in high-intensity psychological treatment, such as cognitive behavior therapy (CBT) and psychodynamic psychotherapy [6]. According to a US survey of psychiatry residency training, high-intensity psychological treatment training receives more didactic and supervision time than low-intensity psychological treatment training [7]. The Japanese Society of Psychiatry and Neurology recommends the following targets for early-career psychiatrists in Japan: acquire basic psychological skills (e.g., build positive relationships with patients and their families), conduct low-intensity psychological treatment (e.g., supportive therapy), and understand high-intensity psychological treatments (e.g., CBT, psychodynamic psychotherapy) [8]. In comparison to the US, greater emphasis is placed on the acquisition of basic psychological skills to conduct low-intensity psychological treatment; moreover, these targets are neither operationalized nor mandated across residency programs. The quantity and quality of psychological treatment training largely depends on the individual resident’s interests and motivations to seek external resources [9]. New training models to increase competency through clinical supervision are now being considered in Japan [10].

The purpose of this exploratory study was to compare early-career psychiatrists in the US and Japan on their initial selection of low- vs. high-intensity psychological treatment for the management of mild to moderate MDD. Bower and Gilbody [11] define treatment intensity as the amount of specialized therapist time required. In the present study, the low-intensity psychological treatment included supportive therapy, active monitoring, and active listening; the high-intensity psychological treatment included CBT, interpersonal psychotherapy, and psychodynamic psychotherapy. By examining early-career psychiatrists’ decisions on which psychological treatments to provide for patients with MDD, we aimed to better understand the global issue of how to disseminate and implement psychological treatment in clinical practice across cultures.

## Methods

We evaluated a sample of 120 early-career psychiatrists who have either completed residency within the past 6 years or are current residents in two selected programs in Japan and the US. Each program consists of 60 residents and operates within large academic teaching hospitals in urban areas. The Japanese residency program includes 2 years of general training (postgraduate year (PGY) 1–2) and 4 years of psychiatric training (PGY 3–6) following 6 years of medical school, whereas the US residency program spans 4 years (PGY 1–4) following 4 years of medical school. Both residency curricula include rotations in outpatient and inpatient services in the institutions’ teaching hospitals, other general hospitals, specialized clinics, and community mental health centers. In addition to treating patients, both groups participate in didactic seminars, conferences, and research. Early-career psychiatrists were selected because their treatment decisions may reflect their education and formal training more saliently than would be the case for senior psychiatrists with years of clinical experience. All participants had the standard workloads of psychiatrists in their countries.

Between January and February of 2014, participants received e-mail invitations to complete brief online surveys about depression treatment. Participation was voluntary and anonymous. Those interested in participating read an electronic consent form detailing risks and benefits of the study. Those who provided informed consent by checking the relevant box on the webpage were able to initiate the survey. Participants who completed the survey had the option of entering a raffle with a 1 in 3 chance of receiving a $10 gift card. All study procedures were approved by the residency directors of the respective institutions and the Keio University School of Medicine Institutional Review Board.

A 10-min online survey developed for this study was self-administered by participants to evaluate their demographic characteristics, average daily outpatient workload, and selection of the initial intervention for MDD treatment. The survey consisted of two clinical case vignettes and 29 multiple-choice questions. All measures were translated into Japanese and English by two bilingual authors (AW, AN). The face validity and cultural appropriateness of the case vignettes were confirmed by expert clinicians (AN, MS, DM) with over 15 years of clinical experience treating patients with MDD in the US and Japan.

In the first two sections of the survey, the participants’ demographic characteristics (age, sex, year medical degree received) and information on previous and current clinical experience (years of postgraduate training, clinical treatment settings) were collected. The average outpatient workload (daily patient volume, time per patient) was also recorded.

In the main section, participants were asked to select which initial treatment modality they would select for two individuals with mild and moderate MDD, as described in the following case vignettes:

1. Mild MDD
  Ms. K, aged 24, presents with a chief complaint of stress. The patient works as a legal assistant at a law firm. She spends long hours working but frequently makes minor clerical mistakes and feels bad for her coworkers. She is worried about making mistakes at a large conference to be held next month and has difficulty sleeping. She feels a bit better on the weekends, when she does not have to work and can see her friends from time to time.
2. Moderate MDD
  Mr. T, aged 29, married with a 6-month-old baby, was laid off 2 months ago. Although he has been searching for a new workplace, he has been unsuccessful. His wife is very worried about their finances and future. Family conversations are becoming less frequent, and the atmosphere at home is tense, but Mr. T does not feel he can ask his friends or parents for help. He cannot see any way forward and feels hopeless. He feels increasingly guilty and sorry for his wife and daughter. The patient expresses some suicidal ideation (“I wish I could fall asleep and never wake up”) but has no specific suicide plans.

For each case vignette, participants selected an initial treatment modality: low-intensity psychological treatment (i.e., supportive therapy, active monitoring, active listening), high-intensity psychological treatment (i.e., CBT, interpersonal psychotherapy, psychodynamic psychotherapy), pharmacotherapy, or others. Participants were subsequently asked which second-line treatment they would choose if the patient exhibited non-response after 4 weeks of initial pharmacotherapy treatment. The second-line treatment options included: psychotherapy, pharmacotherapy, or hospitalization.

Variables were tested for differences between the Japanese and US psychiatrists using Student’s *t* test or the Mann-Whitney *U* test for continuous variables and the *χ*^2^ test or Fisher’s exact test for categorical variables, at a two-sided *P* < 0.05. We report the responses to the case vignettes as the percentage of participants who selected each treatment modality. We used *χ*^2^ tests to determine the differences between the Japanese and US psychiatrists’ treatment selections. Analyses were done with SPSS 22.0 [12].

## Results

We obtained responses from 39 (65 %) US psychiatrists and 42 (70 %) Japanese psychiatrists. The overall demographic characteristics of the two groups did not differ significantly, although the Japanese group included more men and had longer postgraduate training years. Fewer Japanese psychiatrists had clinical training in child psychiatry services or community health centers (Table 1).

**Table 1:** Demographic characteristics of the Japanese and American psychiatrists (*n* = 81)

Variables	Japanese (*n* = 42)		US (*n* = 39)		Analysis		
	Mean	SD	Mean	SD	*t*	df	*P*
Age (years)	34.0	5.2	32.5	4.7	1.34	73	0.09
Postgraduate years	6.9	2.2	3.3	2.6	6.38	73	<0.001
Outpatient workload							
- Number of patients per day	32.7	12.1	4.4	2.4	14.10	67	<0.001
- Length of consultation per patient (min)	8.3	2.6	36.1	13.4	13.39	67	<0.001
	*N*	%	*N*	%	*χ* ^2^	df	*P*
Males	31	73.8	19	48.7	5.39	1	0.02

We found significant differences between the average outpatient workloads of the two groups. The Japanese psychiatrists saw approximately 33 outpatients per day or 8 times the number seen by their US counterparts (*t* = 14.10, df = 67, *P* < 0.001). The average length of a Japanese outpatient consultation was 8 min, almost 30 min shorter than in the US group (*t* = 13.39, df = 67, *P* < 0.001) (Table 1).

**Fig. 1:**
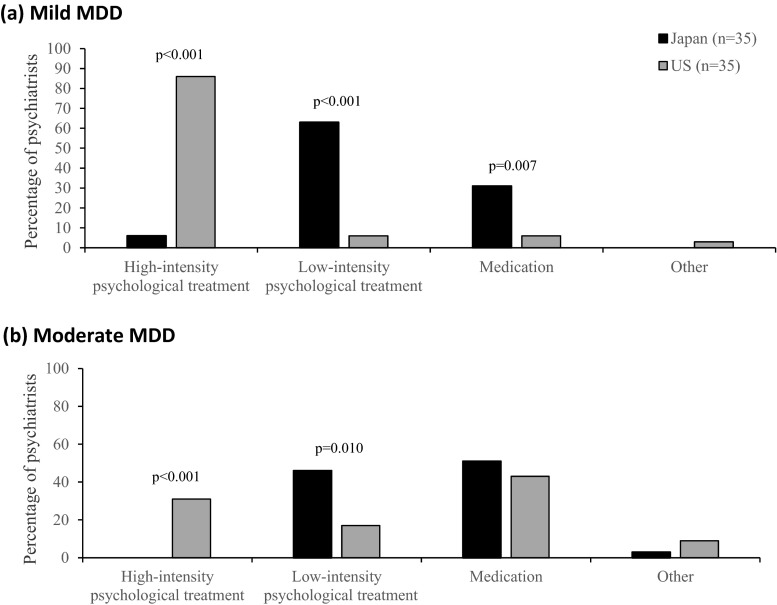
Selection of first-line treatment modality for mild and moderate MDD. **a** Mild MDD. The graph illustrates the distribution of first-line treatment selections for mild MDD between Japanese (*n* = 35) and US (*n* = 35) samples. **b** Moderate MDD. The graph illustrates the distribution of first-line treatment selections for moderate MDD between Japanese (*n* = 35) and US (*n* = 35) samples

For the initial treatment of patients with mild MDD, an overwhelming majority of the US psychiatrists selected high-intensity psychological treatment (US = 30 (86 %) vs. Japan = 2 (6 %), *χ*^2^ = 45.13, *P* < 0.001), whereas the majority of Japanese psychiatrists selected low-intensity psychological treatment (Japan = 22 (63 %) vs. US = 2 (6 %), *χ*^2^ = 25.36, df = 1, *P* < 0.001) (Fig. 1).

For the initial psychological treatment of patients with moderate MDD, about one third of the US psychiatrists selected high-intensity psychological treatment, whereas no Japanese psychiatrist selected this treatment (US = 11 (31 %) vs. Japan = 0 (0 %), *χ*^2^ = 13.05, df = 1, *P* < 0.001). Instead, about half of the Japanese psychiatrists selected low-intensity psychological treatment, whereas less than a fifth of the US psychiatrists chose this option (Japan = 16 (46 %) vs. US = 6 (17 %), *χ*^2^ = 6.63, df = 1, *P* = 0.010) (Fig. 1).

**Fig. 2:**
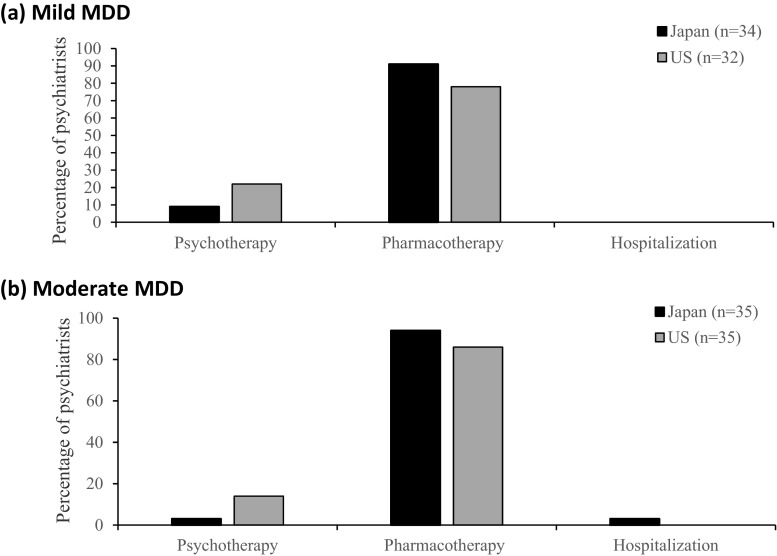
Selection of second-line treatment modality for mild and moderate MDD. **a** Mild MDD. The graph illustrates the distribution of second-line treatment selections for mild MDD between Japanese (*n* = 34) and US (*n* = 32) samples. **b** Moderate MDD. The graph illustrates the distribution of second-line treatment selections for moderate MDD between Japanese (*n* = 35) and US (*n* = 35) samples

When asked about the second-line treatment selection after 4 weeks of non-response to pharmacotherapy, both US and Japanese psychiatrists favored pharmacotherapy, and few selected high-intensity psychological treatment for mild MDD (Japan = 3 (9 %) vs. US = 7 (22 %)) and moderate MDD (Japan = 1 (3 %) vs. US = 5 (14 %)). We found no significant differences between the two groups (Fig. 2).

## Discussion

Overall in this study, early-career US psychiatrists favored high-intensity psychological treatment, whereas Japanese psychiatrists favored low-intensity psychological treatment as the initial approach for patients with mild to moderate MDD. To treat the patient with mild MDD described in the first vignette, the US psychiatrists almost unanimously selected high-intensity psychotherapy, as opposed to less than 10 % of the Japanese psychiatrists. Most of the Japanese psychiatrists instead selected low-intensity psychological treatment, whereas less than 10 % of the US psychiatrists selected this option. In response to the moderate MDD vignette, the difference was less striking, but we found a statistically significant tendency for the US psychiatrists to favor high-intensity psychological treatment and for the Japanese to favor low-intensity treatment.

The striking differences in the mean length and volume of outpatient visits in the two groups highlight the issue of feasibility. Japan has a universal health care system under which almost all inhabitants have insurance and direct access to specialized medical services without having to be seen first by primary care physicians [13]. Within this system, psychiatrists provide first-line services for high volumes of patients with various non-specific symptoms of mild to moderate severity who are normally treated by primary care physicians in other countries. Psychiatrists with medical degrees, not clinical psychologists or licensed clinical social workers, are the primary providers of psychotherapy in Japan, and there is a shortage of providers. With an average of 8 min per patient, the current Japanese system does not allow enough time for selection of or training for high-intensity psychological treatments, such as CBT, which requires 50 min per session. Consequently, low-intensity psychological treatment and pharmacotherapy become the preferred, feasible options. More Japanese than US psychiatrists selected medication to treat both mild and moderate MDD. In Japan, Kasahara’s “minor psychotherapy,” a type of supportive therapy for the initial treatment of depression requiring only 15 min per patient, is widely used [14]. This culture-specific psychological treatment includes components of empathy, accepting attitude, therapeutic alliance, psychoeducation, recommendations for rest, and pharmacotherapy [15] and is recommended in the Japanese Society of Mood Disorders guidelines as the initial treatment for mild MDD [16], indicating its societal acceptance and prevalence.

The distinct emphases on psychological treatment training during residency in the US and Japan may explain the difference between the two groups. As stated earlier, the US residency programs require early-career psychiatrists to achieve competency in supportive therapy, CBT, and psychodynamic psychotherapy. The residents begin training with supportive therapy and subsequently learn CBT and psychodynamic psychotherapy with its unique theories, techniques, and goals for treatment in practice [6]. Although the Japanese residency program follows a similar trajectory, it largely focuses on the primary ability to conduct low-intensity psychological treatment, such as non-directive supportive therapy [8]. Effective doctor-patient communication is associated with patient satisfaction, treatment adherence, and symptom improvement [17]. As such, clinical methods such as therapeutic alliance, clinical interviewing skills, and active listening are viewed as important prerequisites in any approach to psychological treatment and therefore emphasized in Japanese residency programs over supervised clinical experience in high-intensity psychological treatments like CBT. Training in specific psychological treatment models, such as CBT or psychodynamic psychotherapy, is not a requirement for psychiatry residents in Japan [9] and limited in availability, mostly left to the discretion of individual psychiatrists who are strongly interested. Furthermore, long-term psychotherapy treatment is often not feasible in the Japanese residency curricula, where residents rotate at several affiliated teaching hospitals and do not consistently see the same patients. Of note, the Japanese sample had a significantly higher number of postgraduate training years completed and therefore may exhibit some knowledge and skill drift from their original training in comparison to the US sample.

Finally, the differences in training and treatment selections must be considered in relation to the cultural frameworks surrounding psychological treatment in the US and Japan. Western psychological treatment approaches highlight the experience of emotional distress and how it is influenced by patterns of thought and behavior [18], as exemplified by Beck’s cognitive model [19]. In high-intensity psychological treatment such as CBT, patients actively learn to change their thought to improve their emotional state and behavior. Moreover, CBT emphasizes a collaborative therapist-client relationship and teaches patients to be their own therapist. This emphasis may fit appropriately within the individualistic Western culture, which values the autonomous individual [20].

Eastern psychological treatment approaches, in contrast, teach clients to accept emotions and thoughts as transitory and not conducive to conscious control [18]. For example, emotional and physical rest is an important component of both the indigenous Morita therapy [21] and the above-mentioned Kasahara’s minor psychotherapy. During the resting period, clients accept the internal fluctuations of thoughts and feelings, as in the concept of mindfulness. The doctor-patient relationship in Japan, moreover, is often hierarchical [22]. There may be less sociocultural expectation of autonomous choice and decision-making on the part of the patient during treatment. In decisions regarding treatment, patients are more likely to defer to clinicians, whom they consider to be experts. This traditional doctor-patient relationship has been changing since the late 1990s with the adoption of shared decision-making models in the field of Japanese psychiatry, but the change is still recent and gradual [22, 23]. In fact, the national CBT program for treating Japanese patients with depression is more directive and places greater emphasis on problem-solving techniques within interpersonal contexts [24]. Given these culturally influenced concepts of emotion and therapist-client relationship, the relatively universal components of supportive therapy may be more easily translated across cultures, partly explaining the Japanese tendency to prefer low-intensity psychological treatment shown in our study results.

Considering the strong emphasis on biology and neuroscience in psychiatry residency training and the time constraints on psychiatrists, the relatively low selection of medication we saw in this study in comparison to psychological treatment for mild to moderate MDD was unexpected. The selections may be shaped by prescribing guidelines, such as the Maudsley prescribing guidelines [25] or the National Institute for Health and Care Excellence guidelines [26], which do not recommend pharmacotherapy as the first-choice treatment for mild MDD. Moreover, Fournier and colleagues [27] have shown that the benefits of antidepressants over placebos may be minimal or non-existent in patients with mild to moderate MDD. The US residency program represented in our sample offers supportive therapy, psychodynamic training, and CBT, in addition to neuroscience and psychopharmacology, which may explain the relative inclination toward psychological treatments that do not involve medication [28].

This study has several limitations. First, there is a selection bias, and the relatively small sample of participants (*n* = 81) from two psychiatry residency programs may not represent all early-career psychiatrists in the respective countries or worldwide. The US residency program in our sample has a relatively strong psychological treatment component, which could produce a tendency toward selection of psychotherapy. Second, our questionnaire was developed specifically for this study and has not been validated. Third, case vignettes may be interpreted differently from real-life clinical cases, and our findings may not fully reflect actual treatment decisions that psychiatrists make in clinical practice. Fourth, the treatment choices offered to the psychiatrists in this study were broad categories rather than specific psychotherapy models (e.g., CBT) or specific psychotherapeutic components (e.g., therapeutic alliance). Fifth, differences in the health insurance systems of the two countries—which may impact treatment availability and hence treatment selection—were not considered. Despite these limitations, this preliminary analysis demonstrates broad differences between the selections of high- and low-intensity psychological treatments in the US and Japan. Future studies will expand these broader categories into specific components of psychological treatment and compare the treatment selections within larger samples of psychiatrists and clinical psychologists in Western and Eastern countries.

In conclusion, preliminary results showed differences in the rates of selection of high-intensity and low-intensity psychological treatment for mild to moderate MDD made by early-career psychiatrists in the US and Japan. It is often noted that psychological treatment is relatively rare in Asia compared to the West. This study provides a more nuanced account of the possible tendency toward low-intensity psychological treatment in Japan and high-intensity psychological treatment in the US. Feasibility and the nature of training may broadly impact the selection of psychological treatment; moreover, deeply rooted cultural notions about psychological treatments may also affect clinical decisions. In this era of globalization, it is of great interest to continue exploring how to effectively disseminate and implement psychotherapy into clinical practice across multiple cultures.

**Table:** 

Implication for Academic Leaders
• Residency training, availability of psychological treatment modalities, and cultural customs and beliefs may shape treatment selection of these modalities.
• Psychotherapy training for early-career psychiatrists in Japan must be adapted to consider feasibility, including high patient volume and limited patient time, for successful implementation of psychotherapy.
• Psychotherapy training for early-career psychiatrists in Japan may place greater emphasis on directive, interpersonal approaches and components of mindfulness to produce greater cultural fit. Early-career psychiatrists in the US may also adopt these approaches for patients from Japanese or Asian cultures.
